# Trends in Hospitalizations for Alcohol Use Disorder in the US From 1998 to 2016

**DOI:** 10.1001/jamanetworkopen.2020.16580

**Published:** 2020-09-21

**Authors:** Jasvinder A. Singh, John D. Cleveland

**Affiliations:** 1 Division of Rheumatology, Department of Medicine, University of Alabama at Birmingham, Birmingham; 2 Birmingham VA Medical Center, Birmingham, Alabama

## Abstract

This cross-sectional study examines data from the US National Inpatient Sample database for both hospitalization and in-hospital mortality rates of people with alcohol use disorders during the years 1998 to 2016.

## Introduction

Alcohol use disorder (AUD) is among the most prevalent mental disorders worldwide. An estimated 14.5 million people in the US (5%) had an AUD in 2017.^1^ The published US national estimates for AUD hospitalizations are from 2003,^2^ with a lack of contemporary data. Our study objectives were to assess time trends in AUD hospitalizations and associated in-hospital mortality in the US over time. We hypothesized that AUD hospitalizations would increase and associated mortality would decrease over time.

## Methods

This cross-sectional study used data from the US National Inpatient Sample (NIS) database. The NIS is a 20% stratified sample of all US community hospital discharges regardless of the payer type and the largest publicly available all-payer inpatient database in the US.^3^ The University of Alabama at Birmingham’s institutional review board approved this study and waived the need for informed consent because the data are anonymous and publicly available. This study followed the Strengthening the Reporting of Observational Studies in Epidemiology (STROBE) reporting guideline.

This study examined NIS data from the years 1998 to 2016. In 2012, sampling changed from a sample of hospitals to a sample of discharges from all hospitals that participate in the Healthcare Cost and Utilization Project, so new definitions of hospitals and discharges were supplied.

We used the diagnostic codes for AUD in the primary position for hospitalization, excluding codes corresponding to drug or alcohol counseling and rehabilitation or detoxification, as described previously.^4^ We examined time trends in the number and rate of AUD hospitalizations and in-hospital mortality rates during the study period, 1998 to 2016, expressed as per 100 000 NIS claims. Significance of time trend was assessed with the Cochrane-Mantel-Haenszel test. We used the provided set of trend weights up to 2011 and discharge weights from 2012 to 2016 to allow analyses across multiple years, which include the period of design change. We calculated the 95% CI for estimates. A 2-sided *P* < .05 was considered statistically significant. Statistical analysis was performed using SAS statistical software version 9.4 (SAS Institute) from May to December 2019.

## Results

There were a total of 5 590 952 patients with primary AUD hospitalizations. The mean (SE) age was 48.7 (0.04) years. Of these patients, 4 078 733 (73.3%) were men, 3 284 699 (58.9%) were White, 3 155 516 (56.6%) had a comorbidity score (Deyo-Charlson Index score) of 0, and 106 419 (1.9%) died during hospitalization (Table).

**Table.** Cohort Characteristics of People With AUD Hospitalizations^a^

Characteristics	Primary AUD hospitalizations, No. (%)
Age, y	
Mean (SE)	47.8 (0.04)
Median	47.2
Female	1 483 491 (26.7)
Race	
Non-White	2 288 502 (41.1)
White	3 284 699 (58.9)
Deyo-Charlson Index score	
0	3 155 516 (56.6)
≥1	2 418 033 (43.4)
Died during hospitalization	106 419 (1.9)

Abbreviations: AUD, alcohol use disorder; *ICD-9-CM, International Classification of Diseases, Ninth Revision, Clinical Modification; ICD-10-CM, International Statistical Classification of Diseases, Tenth Revision, Clinical Modification*.

^a^ Cases of AUD were defined via the appearance of any of the following *ICD-9-CM* or *ICD-10-CM* codes in the primary position: 291.xx, 303.xx, 305.0x, 357.5, 425.5, 535.30, 535.31, 571.0, 571.1, 571.2, 571.3, E860.0, F10.xxx, G31.2, G62.1, G72.1, I42.6, K29.2xx, P04.3, O35.4xxx, K86.0, T51.0xxx, T51.1xxx, or T51.9xxx. We excluded hospitalization with *ICD-9-CM* or *ICD-10-CM* diagnostic codes 303.03, 303.93, or 305.03, F10.11xx, or F10.21xx or *ICD-9-CM* or *ICD-10-CM* procedure codes 94.45, or 94.64-94.69, HZ2xxxx-3xxxx, HZ4xxxx, HZ5xxxx-6xxxx, HZ81xxx-82xxx, HZ84xxx-86xxx, HZ88xxx-89xxx, HZ91xxx-92xxx, HZ94xxx-96xxx, HZ98xxx-99xxx corresponding to drug or alcohol counseling and rehabilitation or detoxification.

We found a 3.5% increase in the AUD hospitalizations from 274 652 hospitalizations (95% CI, 243 587-305 717 hospitalizations) in 1998 to 284 275 hospitalizations (95% CI, 275 403-293 146 hospitalizations) in 2016; claims decreased first until 2005, and then increased to 2015 (Figure). There was a 25% and 28% decrease in the number of AUD hospitalization deaths and mortality rate per 100 000 total NIS claims, respectively (Figure; lowest in 2012). In-hospital mortality for AUD hospitalizations decreased by 25% (from 7305 [95% CI, 6757-7852] deaths per 100 000 claims in 1998 to 5475 [95% CI, 5088-5861] deaths per 100 000 claims in 2016) compared with a 20% decrease (from 842 386 [95% CI, 808 314-876 458] deaths per 100 000 claims in 1998 to 675 114 [95% CI, 659 158-691 071] deaths per 100 000 claims in 2016) for all other NIS claims.

**Figure.**
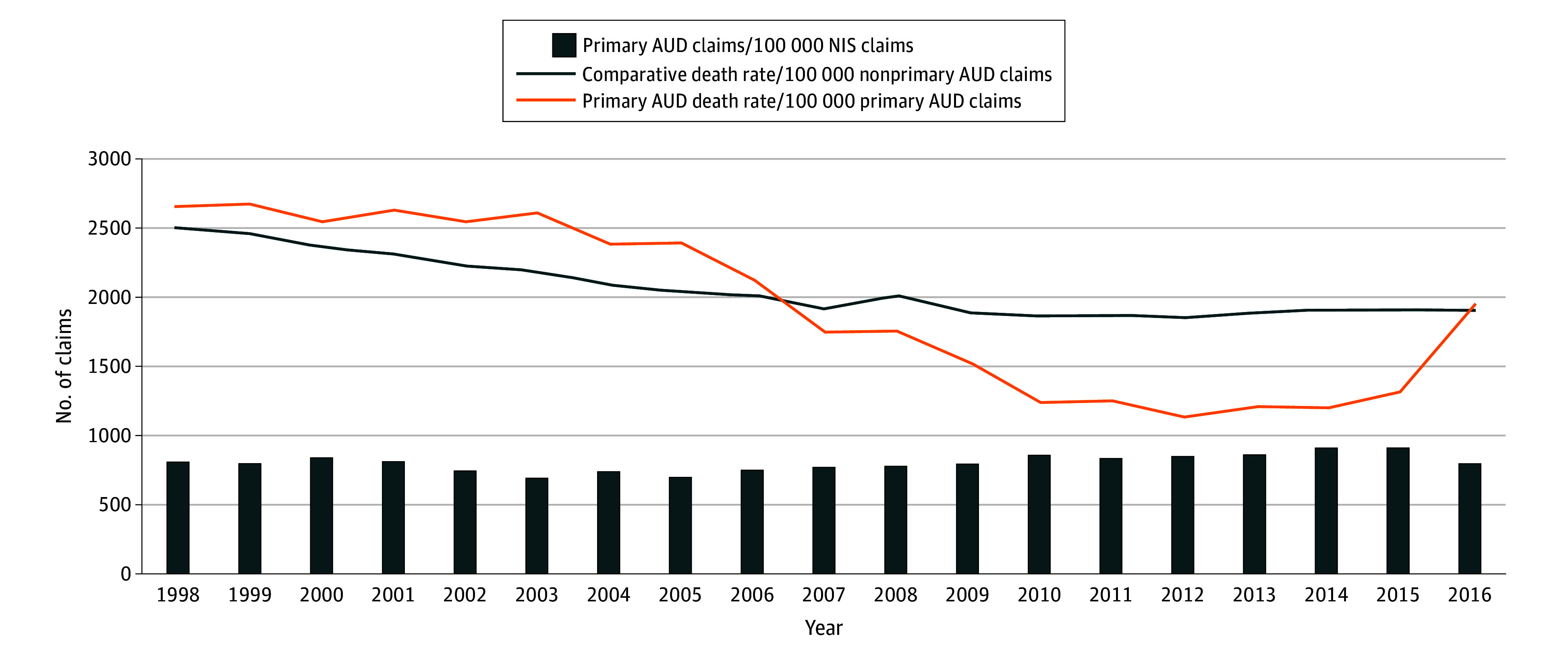
Rate of Occurrence of Primary Alcohol Use Disorder (AUD) Hospitalization Claims and Associated Mortality Compared With Mortality for Nonprimary AUD Hospitalizations Over Time The x-axis shows each calendar year during the study period, and the y-axis shows the number of claims for the respective outcome. Bars show the rate of primary AUD claims per 100 000 total NIS claims. The orange line shows the mortality rate in primary AUD hospitalizations per 100 000 primary AUD claims. The dark blue line shows the comparative mortality rate in nonprimary AUD hospitalizations per 100 000 nonprimary AUD hospitalization claims. NIS indicates US National Inpatient Sample.

## Discussion

In this cross-sectional study, we found a 28% decrease in in-hospital mortality rate per 100 000 total NIS claims from 1998 to 2016 among people with AUD hospitalizations. AUD hospitalization mortality reduction might be attributable to prompt recognition and treatment of AUD-associated medical complications^5^ and an integrated care model for mental health services^6^ in the more recent years. There was 3.5% increase in the rate of AUD hospitalizations from 1998 to 2016, showing a decline first until 2005 then an increase through 2015. Although AUD hospitalizations increased minimally, the overall health care impact of AUD is substantial.^1^ Both of these findings were consistent with our a priori hypotheses.

This study had some limitations that need to be addressed. We only examined hospitalizations with a primary diagnosis of AUD. NIS counts hospitalizations, not people, and excludes military or Veterans Affairs hospitals. Misclassification bias is possible because of the use of diagnostic codes for AUD.

From 1998 to 2016 in the US, AUD hospitalizations increased slightly while in-hospital mortality for patients hospitalized with AUD decreased significantly. A better understanding of what causes these time trends could help further improve AUD hospitalization outcomes and reduce mortality.

## References

[1] Substance Abuse and Mental Health Services Administration. Key substance use and mental health indicators in the United States: results from the 2017 National Survey on Drug Use and Health. Published 2018. Accessed November 18, 2019. https://www.samhsa.gov/data/sites/default/files/cbhsq-reports/NSDUHFFR2017/NSDUHFFR2017.htm 33661590

[2] Russo CA, Elixhauser A; Agency for Healthcare Research and Quality Healthcare Cost and Utilization Project (HCUP). Statistical brief #4: hospitalizations for alcohol abuse disorders, 2003. Published 2006. Accessed August 19, 2020. https://www.hcup-us.ahrq.gov/reports/statbriefs/sb4.jsp 21938847

[3] Agency for Healthcare Research and Quality, Healthcare Cost and Utilization Project (HCUP). NIS overview. Published 2012. Accessed May 6, 2020. https://www.hcup-us.ahrq.gov/nisoverview.jsp 21413206

[4] Heslin KC, Elixhauser A, Steiner CA; Agency for Healthcare Research and Quality, Healthcare Cost and Utilization Project (HCUP). Statistical brief #191: hospitalizations involving mental and substance use disorders among dults, 2012. Published June 2015. Accessed August 19, 2020. https://www.hcup-us.ahrq.gov/reports/statbriefs/sb191-Hospitalization-Mental-Substance-Use-Disorders-2012.jsp 26290939

[5] Hilton ME, Maisto SA, Conigliaro J, . Improving alcoholism treatment across the spectrum of services. Alcohol Clin Exp Res. 2001;25(1):128-135. doi:10.1111/j.1530-0277.2001.tb02137.x 11198708

[6] Bartels SJ, Coakley EH, Zubritsky C, ; PRISM-E Investigators. Improving access to geriatric mental health services: a randomized trial comparing treatment engagement with integrated versus enhanced referral care for depression, anxiety, and at-risk alcohol use. Am J Psychiatry. 2004;161(8):1455-1462. doi:10.1176/appi.ajp.161.8.1455 15285973

